# Acupuncture protects against cerebral ischemia–reperfusion injury via suppressing endoplasmic reticulum stress-mediated autophagy and apoptosis

**DOI:** 10.1186/s10020-020-00236-5

**Published:** 2020-11-10

**Authors:** Xiaowei Sun, Hao Liu, Zhongren Sun, Beng Zhang, Xinyu Wang, Tingting Liu, Tingting Pan, Ying Gao, Xicheng Jiang, Hongtao Li

**Affiliations:** 1 Department of Acupuncture and Moxibustion, The First Affiliated Hospital, Heilongjiang University of Chinese Medicine, Harbin, 150040 Heilongjiang People’s Republic of China; 2 Department of Acupuncture and Moxibustion, Tongde Hospital of Zhejiang Province, Zhejiang Institute of Traditional Chinese Medicine, Hangzhou, 310012 Zhejiang People’s Republic of China; 3 Key Laboratory of Acupuncture Clinical Neurobiology (Encephalopathy), Heilongjiang University of Chinese Medicine, Harbin, 150040 Heilongjiang People’s Republic of China; 4 Department of Acupuncture and Moxibustion, Shenzhen Hospital, Beijing University of Chinese Medicine, Shenzhen, People’s Republic of China; 5 Graduate School, Heilongjiang University of Chinese Medicine, Harbin, 150040 Heilongjiang People’s Republic of China; 6 Department of Synopsis of the Golden Chamber, School of Basic Medical Sciences, Heilongjiang University of Chinese Medicine, 24 Heping Road, Harbin, 150040 People’s Republic of China; 7 Department of Orthopaedics and Traumatology, The First Affiliated Hospital, Heilongjiang University of Chinese Medicine, 26 Heping Road, Harbin, 150040 Heilongjiang People’s Republic of China

**Keywords:** Acupuncture, Cerebral I/R injury, ER stress, Autophagy, Apoptosis

## Abstract

**Background:**

Acupuncture treatment possesses the neuroprotection potential to attenuate cerebral ischemia–reperfusion (I/R) injury. Endoplasmic reticulum (ER) stress has been suggested to be involved in the pathogenic mechanism of cerebral I/R injury. Whether acupuncture protects against cerebral I/R injury via regulating ER stress remains unclear. This study aimed to evaluate the role of ER stress in the neuroprotection of acupuncture against cerebral I/R injury and its underlying mechanisms.

**Methods:**

Cerebral I/R injury was induced by middle cerebral artery occlusion (MCAO) in rats. Acupuncture was carried out at Baihui (GV 20), and Qubin (GB7) acupoints in rats immediately after reperfusion. The infarct volumes, neurological score, ER stress, autophagy and apoptosis were determined.

**Results:**

Acupuncture treatment decreased infarct volume, neurological score and suppressed ER stress via inactivation of ATF-6, PERK, and IRE1 pathways in MCAO rats. Attributing to ER stress suppression, 4-PBA (ER stress inhibitor) promoted the beneficial effect of acupuncture against cerebral I/R injury. Whereas, ER stress activator tunicamycin significantly counteracted the neuroprotective effects of acupuncture. In addition, acupuncture restrained autophagy via regulating ER stress in MCAO rats. Finally, ER stress took part in the neuroprotective effect of acupuncture against apoptosis in cerebral I/R injury.

**Conclusions:**

Our findings suggest that acupuncture offers neuroprotection against cerebral I/R injury, which is attributed to repressing ER stress-mediated autophagy and apoptosis.

## Introduction

Ischemic stroke is a major type of stroke, which results in death and long-term disability worldwide (Behdarvandy et al. 2019). Rapid restoration of cerebral blood flow is the preferred treatment method for ischemic stroke (Schregel et al. 2018). However, the ischemic brain tissue reperfusion injury may subsequently occur and greatly influent the prognosis (Sun et al. 2018). Therefore, searching effective therapeutic strategy for cerebral ischemic–reperfusion (I/R) injury is extremely important to ischemic stroke. Acupuncture is a significant therapeutic tool of traditional Chinese medicine, which has gained wide acceptance around the world, because of its convenience, safety, and effectiveness (Zhuang et al. 2013). Growing evidence has demonstrated that acupuncture could attenuate cerebral I/R injury through repressing oxidative stress injury, inflammation, apoptosis, autophagy, and so on (Chen et al. 2016; Siu et al. 2004; Wu et al. 2015). Still, the protective mechanisms of acupuncture need to be elaborated.

It is reported that cerebral I/R injury may lead to the aggregation of unfolded or misfolded proteins in endoplasmic reticulum (ER) and trigger ER stress (Ahsan et al. 2019; Gong et al. 2017). Unfolded protein response (UPR) is activated by ER stress, thus the biomarkers for UPR can indicate the occurrence of ER stress. PKR-like ER kinase (PERK), inositol-requiring protein 1 (IRE1), and activating transcription factor 6 (ATF-6) are three UPR signaling arms (Clarke et al. 2012). Glucose-regulated protein 78 (GRP78) is an important modulator of UPR. In addition, the severe and continued ER stress may result in increased transcription of CHOP, an essential molecule in the apoptotic signaling pathway, and then trigger apoptosis (Tsai et al. 2018). Previous studies suggested that attenuating ER stress could effectively relieve cerebral I/R injury (Feng et al. 2019; Nan et al. 2019).

Autophagy is a biological process specific for eukaryotic cell and essential to maintain normal function of cells. A larger number of experiments have demonstrated that autophagy is involved in the pathogenesis of cerebral I/R injury (Luo et al. 2017; Wang et al. 2018). Interestingly, ATF-4 as a downstream effector of PERK, can induce the expression of autophagy-related genes, which links ER stress with autophagy (B'Chir et al. 2013). A recent study suggest that the close connection between ER stress and autophagy plays pivotal roles in the progression of cerebral I/R injury (Zhang et al. 2014). Acupuncture has been shown to protect against heroin addiction-induced brain injury via regulating ER stress (Gao et al. 2017). However, whether acupuncture can alleviate cerebral I/R injury through affecting ER stress-dependent autophagy has not been determined.

In the present study, the cerebral I/R injury model was established by middle cerebral artery occlusion (MCAO) in rats. The effects of acupuncture on ER stress and its dependent autophagy and apoptosis were investigated in cerebral I/R injury. Our results further elucidate the mechanisms of acupuncture promoting functional recovery after ischemic stroke.

## Methods

### Animal model

**Fig. 1:**
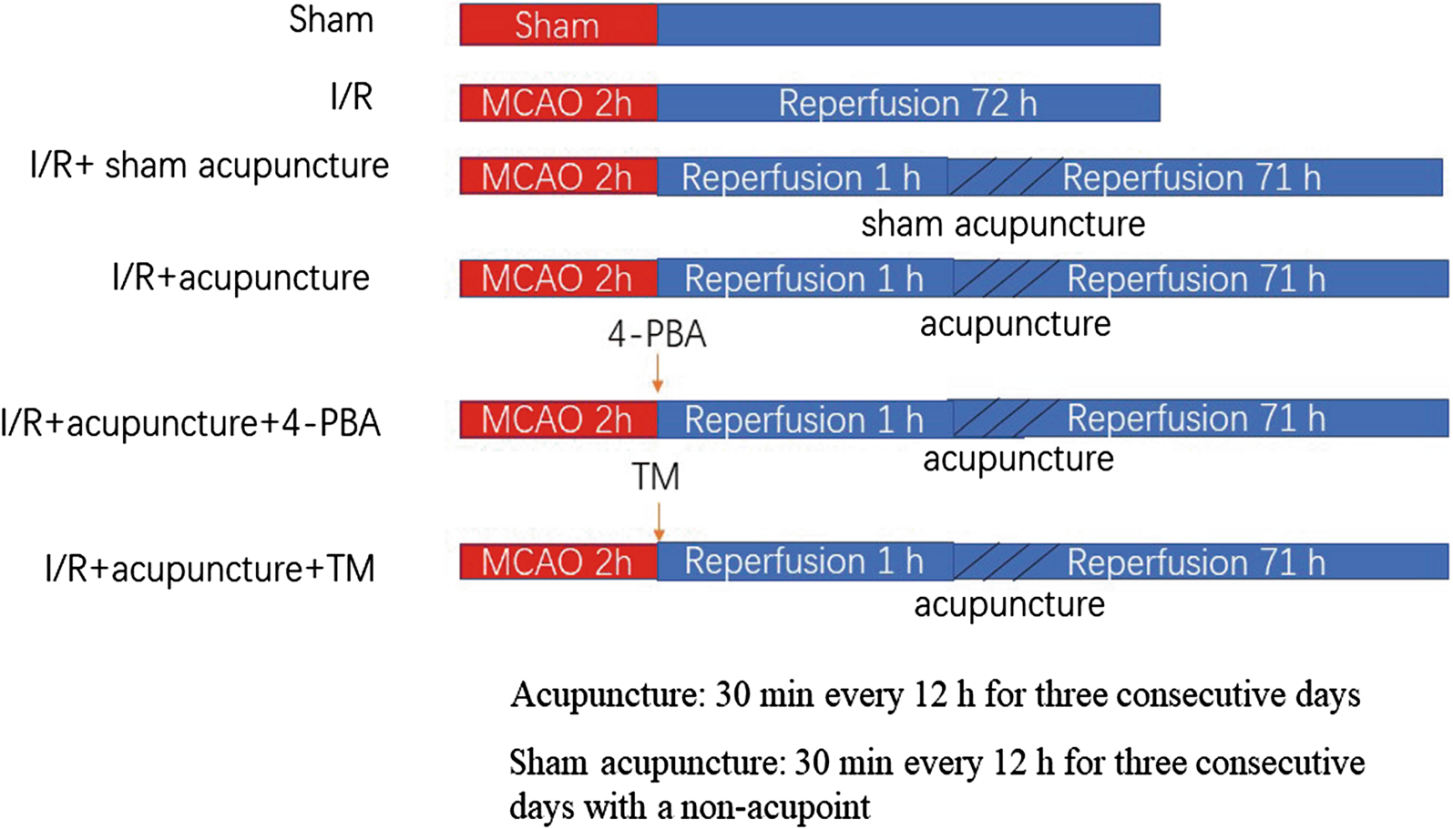
The experimental protocol for different groups

Male Sprague-Dawley rats were obtained from Changsheng biotechnology co., Ltd (Benxi, Liaoning, China) and adaptively raised for one week. Subsequently, the rats were assigned to the following four groups at random (n = 24 per group): sham, cerebral I/R, cerebral I/R+ sham acupuncture, and cerebral I/R+ acupuncture. We performed MCAO to induce cerebral I/R injury. Briefly, rats were exposure to anesthesia and fixed in the supine position. Then the right common carotid artery (CCA), external carotid artery (ECA) and internal carotid artery (ICA) were exposed and the CCA and ECA were ligated. After that we inserted a nylon suture of diameter 0.285 mm into the ICA from the incision on CCA and moved forward for about 18 mm to block middle cerebral artery for 120 min. Reperfusion was carried out by drawing out the nylon suture. The sham rats were received surgical operation in the same way but without blocking middle cerebral artery. After the reperfusion for 1 h, the rats in cerebral I/R+ acupuncture group were received electroacupuncture (Baihui (DU 20) and Qubin (GB7)). The entry point was GV20, and the needle traveled subcutaneously to GB7. Acupuncture was conducted using 0.3 mm × 25 mm needles with inserting depth of 0.5 cm at 2–20 Hz for 30 min every 12 h for three consecutive days. While the rats in cerebral I/R+ sham acupuncture group were subjected to the same electroacupuncture procedure but received electroacupuncture with a non-acupoint (from 1 cm to the right of GV20, 1.5 cm towards the nose). The experimental protocol was shown in Fig. 1.

### Inhibition or activation of ER stress in rats

The inhibitor of ER stress 4-PBA (10 mg/kg, 10 μL, Aladdin, Shanghai, China) or activator of ER stress tunicamycin (TM, 25 μM, 10 μL, Meilunbio, Dalian, China) was injected into the right ventricle of rats at once after the MCAO. The other group rats were received ventricle injection with vehicle in the same volume.

### Neurobehavioral evaluation

Neurobehavioral evaluation was used to assess neural functional injury at 72 h after the reperfusion as previously described (Zhao et al. 2019). There are four individual tests: movement experiment, sensory experiment, beam balance test, and reflex and abnormal movement experiment. The score ranges from 0 to 18. The higher neurological score indicates more severe neural functional injury.

### TTC staining for infarct volume detection

The brain tissues were collected at 72 h after the reperfusion, kept at – 20 ℃ for 2 h and then cut into five 2-mm slices. After staining in 0.4% TTC solution (Sangon Biotech, Shanghai, China) for 10–15 min at 37 ℃, the slices were photographed.

### Brain water content

The obtained brains were weighed as wet weight. Then the brains were put into a 100 ℃ oven for 72 h and weighed as dry weight. The water content was calculated as follow: (wet weight − dry weight)/wet weight × 100%.

### Evans blue (EB) staining

The blood brain barrier leakage was assessed by EB staining. Briefly, after reperfusion for 72 h, 2% EB in saline (2 mg/kg) was intravenously injected via a femoral vein. 1 h after the injection of EB, the rats were transcardially perfused with heparinized saline. After decapitation, the brains were removed and separated into two hemispheres along the sagittal suture.

### Western blotting analysis

Cell lysis buffer (Beyotime, Haimen, Jiangsu, China) added with 1 mM PMSF was prepared for total protein isolation from brain tissues. BCA Protein Assay Kit (Beyotime) was selected for protein concentration determination. Then 20 μL same amount of protein samples were electrophoresed on polyacrylamide gel and transferred onto PVDF membranes (Millipore, USA). 5% skimmed milk in tris buffered saline tween buffer was adopted for blocking. After that, primary antibodies against GRP78 (1:5000, Proteintech, Wuhan, China), ATF-6 (1:1000, Proteintech), p-PERK (1:1000, Cell Signaling Technology, USA), PERK (1:1000, Cell Signaling Technology), p-IRE1 (1:1000, Abcam, UK), IRE1 (1:1000, Abcam), p-eIF2α (1:500, Abcam), eIF2α (1:1000, Abcam), ATF-4 (1:1000, Proteintech), CHOP (1:500, Proteintech), Beclin-1 (1:1000, Proteintech), LC3I/II (1:1000, Proteintech), p62 (1:2000, Proteintech), Bcl-2 (1:5000, Proteintech), Bax (1:2000, Proteintech), cleaved caspase-12 (1:1000, Bioss, Beijing, China), cleaved caspase-9 (1:1000, Affinity Biosciences, USA), cleaved caspase-3 (1:1000, Cell Signaling Technology), cleaved PARP (1:1000, Cell Signaling Technology), or β-actin (1:1000, Santa Cruz, USA) were applied at 4 ℃ overnight. The corresponding secondary antibodies were used for incubation for 45 min at 37 ℃. The results were acquired after reaction with ECL reagent (Beyotime) and exposure in a dark room.

### Immunofluorescence staining

The paraffin-embedded brain tissues were cut into 5-μm sections. Then the sections were immersed in xylene for dewaxing, and dipped in 95%, 85%, and 75% ethanol, respectively. After receiving microwave antigen retrieval and blocking in goat serum, the sections were incubated with primary antibodies ATF-6 (1:200, Proteintech), p-PERK (1:200, Bioss), p-IRE1 (1:200, Novus, USA), or LC3 (1:200, Proteintech) in a humidified box at 4 ℃ overnight. Then Cy3-labeled secondary antibody (1:200, Beyotime) was added for 60 min at room temperature, followed by nuclear counterstaining with DAPI. The sections were observed and photographed under a fluorescent microscope (Olympus, Japan).

### Terminal deoxynucleotidyl transferase-mediated nick end labeling (TUNEL) assay

To determine apoptosis in brain tissues, the above prepared sections of brain were subjected to TUNEL using the In Situ Cell Death Detection Kit (Roche, Switzerland) following the manual step.

### Statistical analysis

The experimental data are presented as mean ± standard deviation and analyzed using GraphPad Prism 8.0 software. One-way analysis of variance followed by Newman–Keuls post hoc test was performed for multiple group comparison. Neurological score was analyzed using Kruskal–Wallis test followed by Dunn’s test. P < 0.05 was regarded as statistical significance.

## Results

### Effect of acupuncture on cerebral I/R injury

**Fig. 2:**
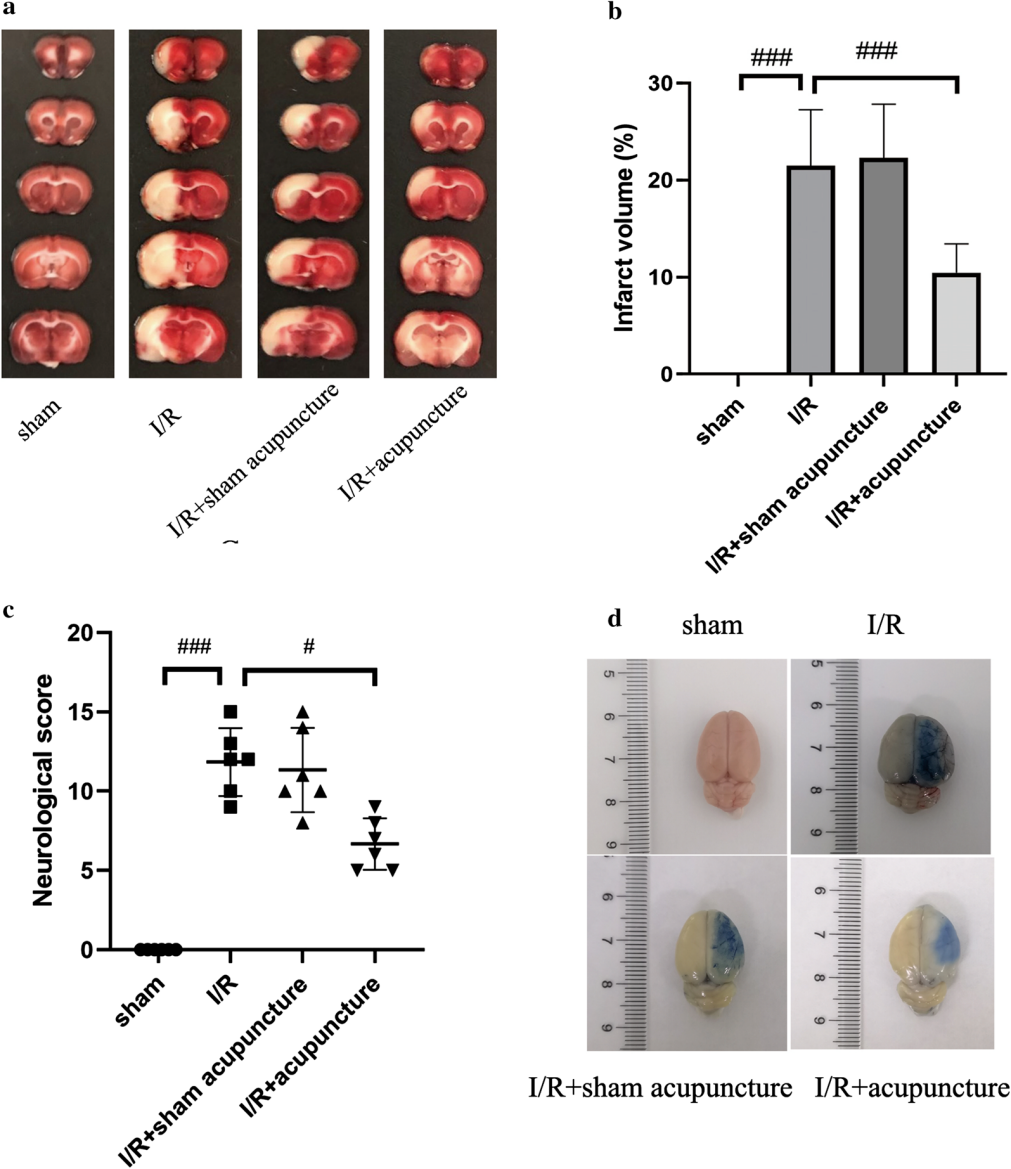
Effect of acupuncture on cerebral ischemia–reperfusion (I/R) injury. **a**, **b** TTC staining was performed to determine the infarct volume of rats at 72 h after reperfusion in the four groups (sham, I/R, I/R+ sham acupuncture, I/R+ acupuncture). Brain infarct volume presented as a percentage of the infarct hemisphere. **c** The neurological deficits were evaluated by neurological score at 72 h after reperfusion. Neurological scores are presented as median (interquartile range). Results represent at least three independent experiments. **d** Blood brain barrier leakage was detected by Evans blue staining. Each experimental result was presented as mean ± standard deviation (n = 6). ^#^P < 0.05, ^###^P < 0.001 versus the indicated group

The beneficial effect of acupuncture against cerebral I/R injury was evaluated firstly. As shown in Fig. 2a, b, TTC staining results demonstrated that the infarct volume was increased by MCAO, which was effectively reduced by acupuncture treatment. Moreover, the neurological score surged significantly in I/R injury group, suggesting severe neural functional injury. Acupuncture treatment could alleviate neural functional injury as evidenced by decreased neurological score (Fig. 2c). As shown in Fig. 2d, BBB leakage assessed by EB staining was restrained by acupuncture treatment. However, sham acupuncture did not show any neuroprotection in cerebral I/R injury. These findings indicated that acupuncture treatment could relieve cerebral I/R injury in rats.

### Effect of acupuncture on cerebral I/R injury-induced ER stress

**Fig. 3:**
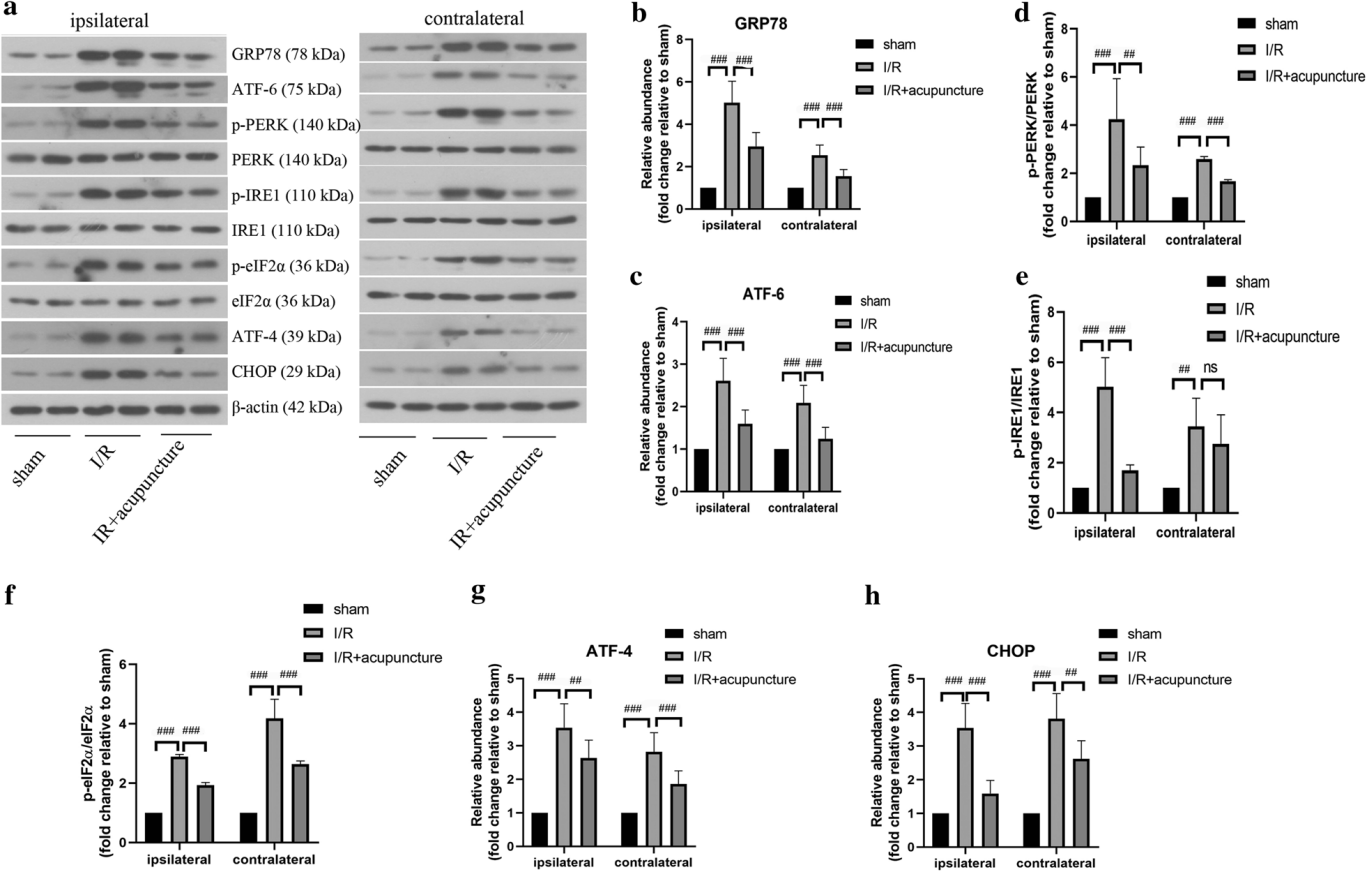
Effect of acupuncture on cerebral ischemia–reperfusion (I/R) injury-induced endoplasmic reticulum (ER) stress. Experiments were performed at 72 h after the reperfusion in the ipsilateral and contralateral brain tissues. **a** The protein levels of glucose-regulated protein 78 (GRP78), transcription factor 6 (ATF-6), PKR-like ER kinase (PERK), phosphorylated PERK (p-PERK), inositol-requiring protein 1 (IRE1), phosphorylated IRE1 (p-IRE1), α unit of eukaryotic initiation factor 2 (eIF2α), phosphorylated eIF2α (p-eIF2α), transcription factor 4 (ATF-4), and C/EBP-homologous protein (CHOP) in brain tissues were detected by western blotting assay. β-actin was used as an internal control. **b**–**h** The relative abundance of the protein bands was calculated and shown. Results represent at least three independent experiments. Each experimental result was presented as mean ± standard deviation (n = 6). ^##^P < 0.01, ^###^P < 0.001 versus the indicated group

**Fig. 4:**
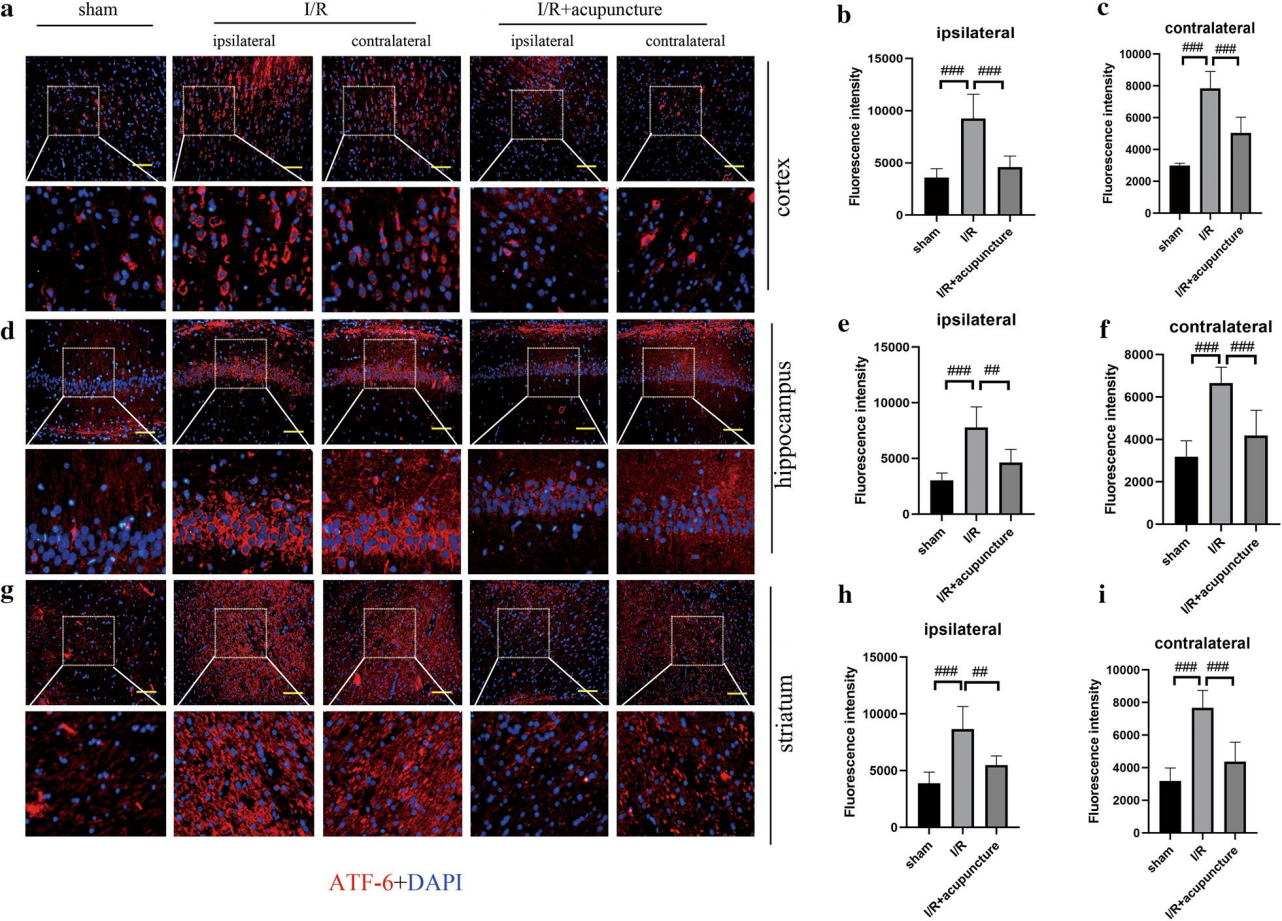
Acupuncture repressed cerebral ischemia–reperfusion (I/R) injury-induced endoplasmic reticulum (ER) stress via inhibiting transcription factor 6 (ATF-6) expression. Immunofluorescence analysis of ATF-6 expression (red) in cortex (**a**–**c**), hippocampus (**d**–**f**), and striatum (**g**–**i**) of contralateral and ipsilateral brains at 72 h after reperfusion. The nuclei were counterstained with DAPI (blue). Scale bars, 100 μm. Results represent at least three independent experiments. Each experimental result was presented as mean ± standard deviation (n = 6). ^##^P < 0.01, ^###^P < 0.001 versus the indicated group

**Fig. 5:**
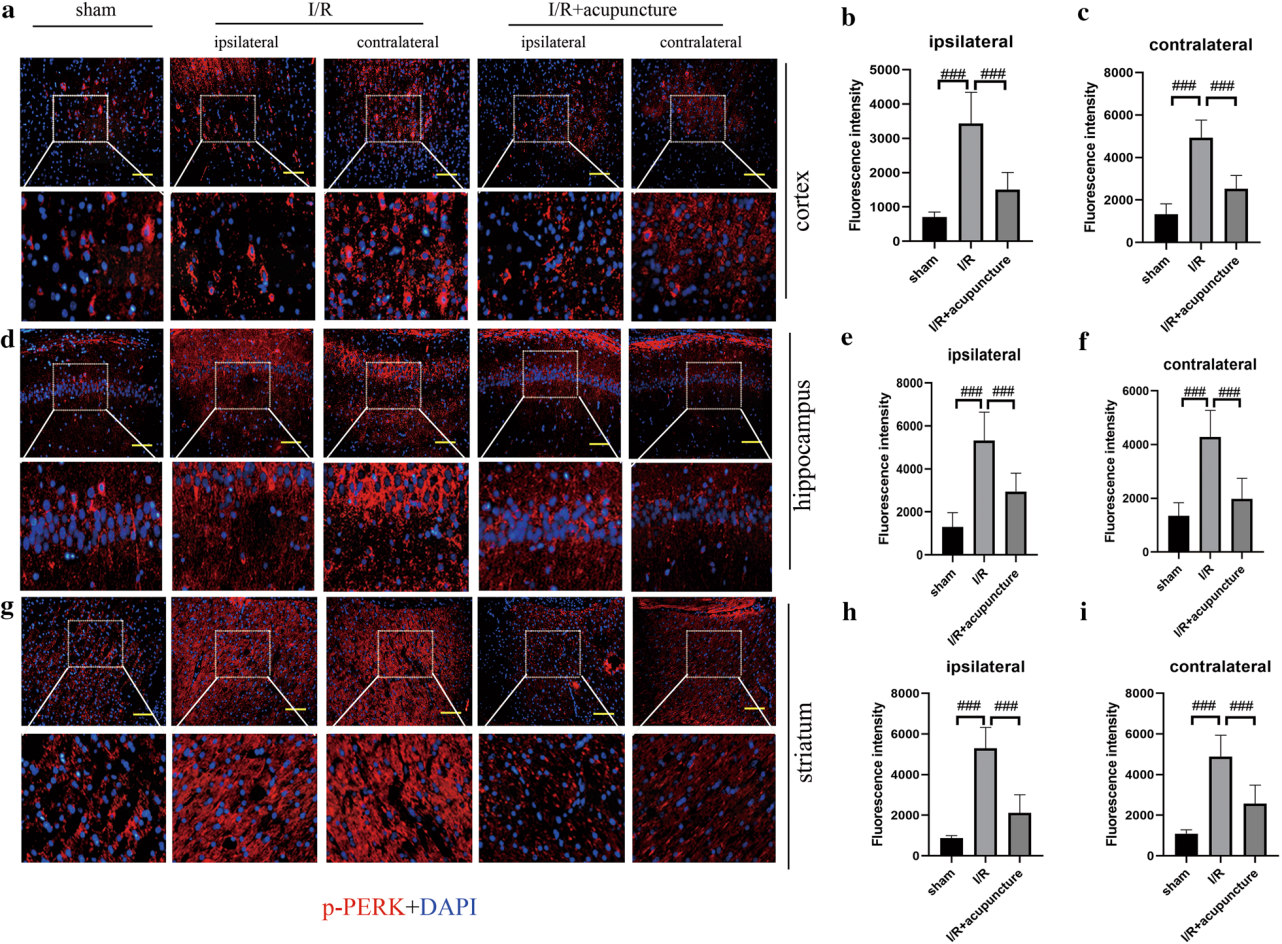
Acupuncture repressed cerebral ischemia–reperfusion (I/R) injury-induced endoplasmic reticulum (ER) stress via inhibiting phosphorylated PKR-like ER kinase (p-PERK) expression. The expression of p-PERK (red) in cortex (**a**–**c**), hippocampus (**d**–**f**), and striatum (**g**–**i**) of contralateral and ipsilateral brains at 72 h after reperfusion was detected by immunofluorescence analysis. The nuclei were counterstained with DAPI (blue). Scale bars, 100 μm. Results represent at least three independent experiments. Each experimental result was presented as mean ± standard deviation (n = 6). ^###^P < 0.001 versus the indicated group

**Fig. 6:**
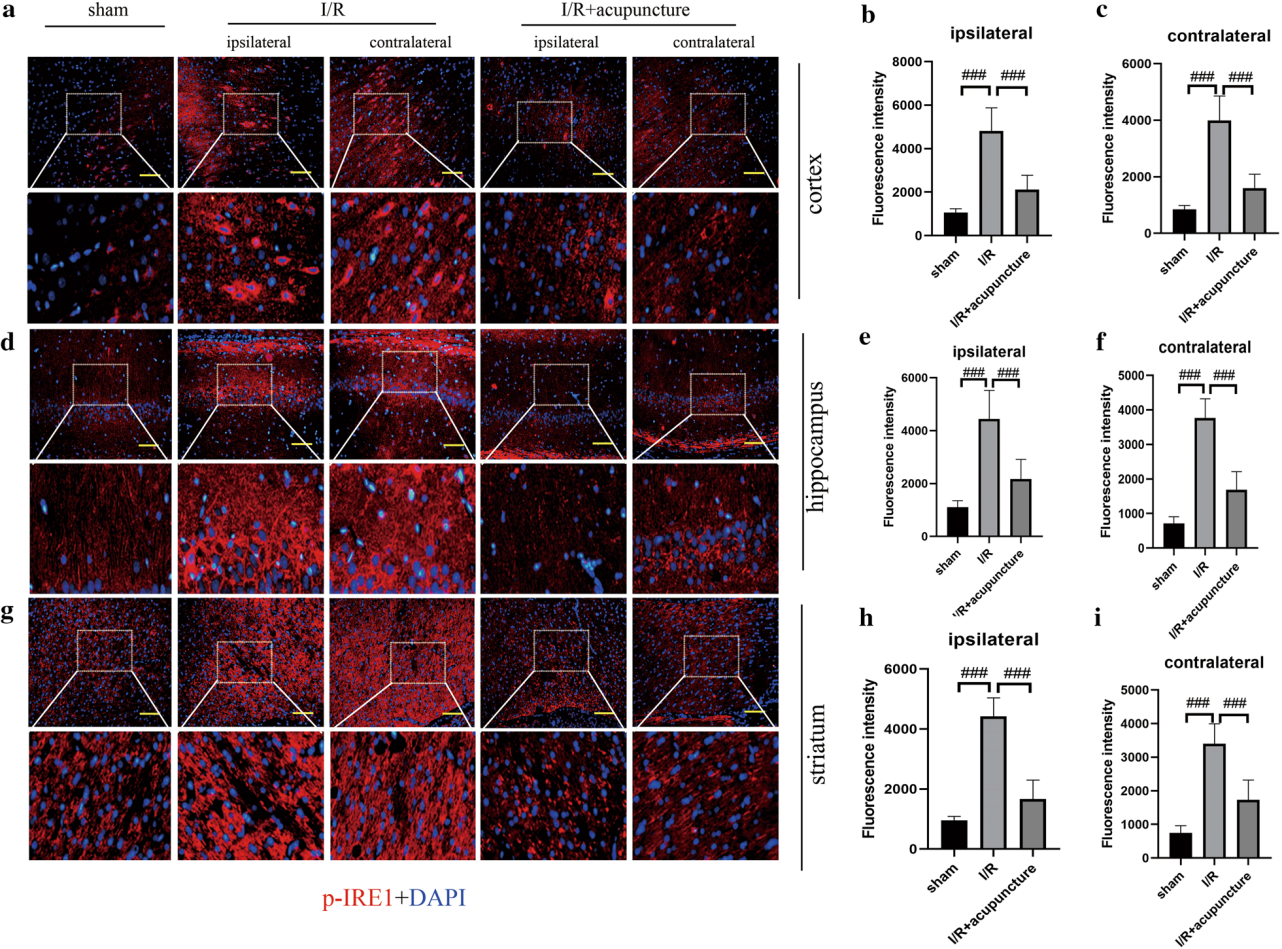
Acupuncture repressed cerebral ischemia–reperfusion (I/R) injury-induced endoplasmic reticulum (ER) stress via inhibiting phosphorylated inositol-requiring protein 1 (p-IRE1) expression. The expression of p-IRE1 (red) in cortex (**a**–**c**), hippocampus (**d**–**f**), and striatum (**g**–**i**) regions of contralateral and ipsilateral brains at 72 h after reperfusion was assessed by immunofluorescence analysis. The nuclei were counterstained with DAPI (blue). Scale bars, 100 μm. Results represent at least three independent experiments. Results represent at least three independent experiments. Each experimental result was presented as mean ± standard deviation (n = 6). ^###^P < 0.001 versus the indicated group

To further probe the protective mechanisms of acupuncture in cerebral I/R injury, we focused on ER stress. As presented in Fig. 3a–h, obvious increases in protein levels of GRP78, ATF-6, ATF-4, CHOP and rations of p-PERK/PERK, p-IRE1/IRE1, and p-eIF2α/eIF2α were observed in both ipsilateral and contralateral cerebral hemispheres of rats compared with sham group. However, treatment with acupuncture remarkably restrained the above changes. In addition, immunofluorescent staining for ATF-6, p-PERK, and p-IRE1 in ipsilateral and contralateral cerebral hemisphere regions of cortex, hippocampus and striatum was carried out. As illustrated in Figs. 4, 5 and 6, the expression of ATF-6, p-PERK, and p-IRE1 in cortex, hippocampus and striatum of ipsilateral and contralateral cerebral hemisphere was significantly enhanced in cerebral I/R injury group compared with that in sham group, which was obviously suppressed by acupuncture treatment. However, the levels of ATF-6, p-PERK, and p-IRE1 were not affected by sham acupuncture (Additional file 1: Fig. S1). The above results suggested that acupuncture inhibited cerebral I/R injury-induced ER stress.

### Acupuncture relieved cerebral I/R injury via suppressing ER stress

**Fig. 7:**
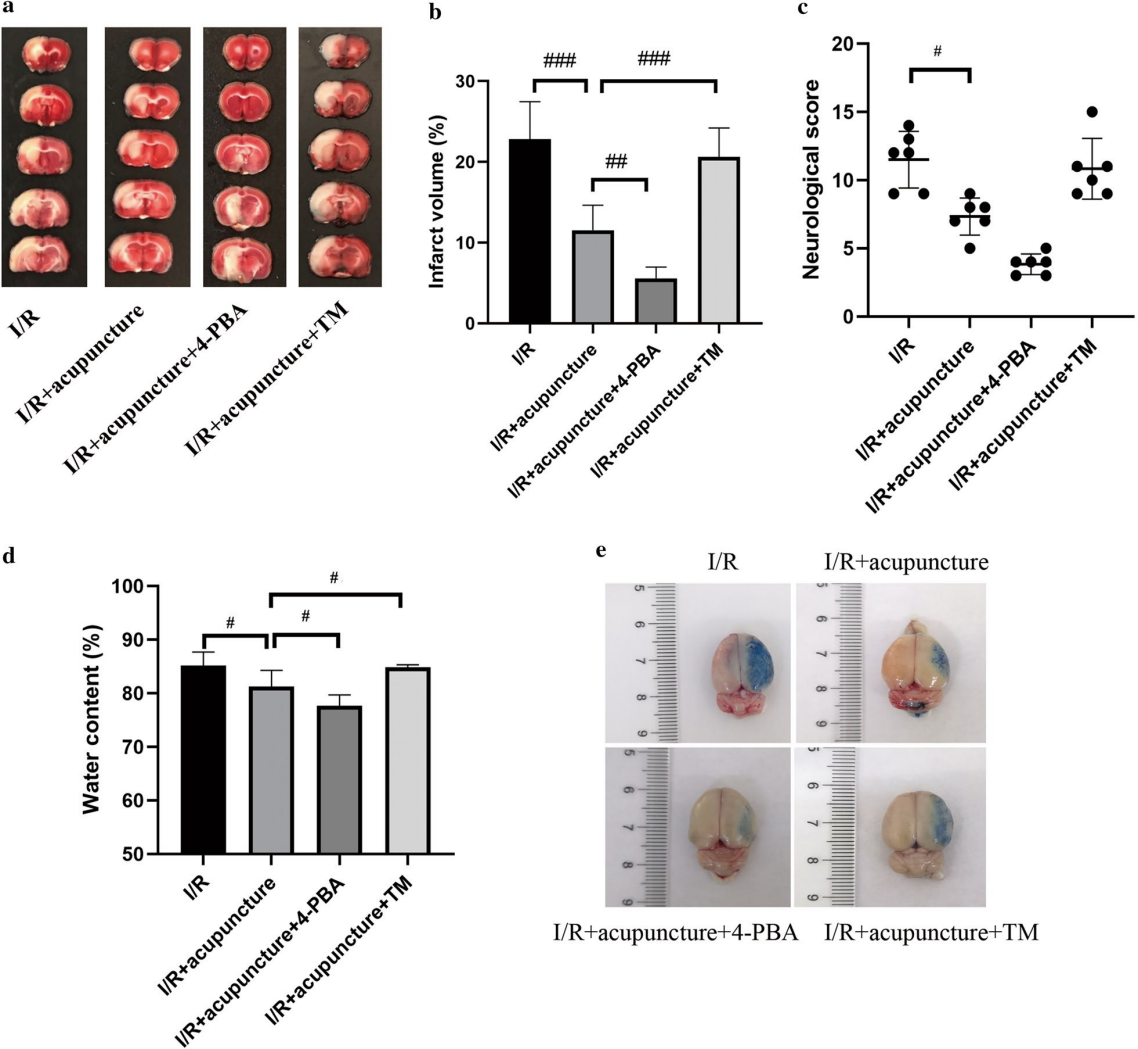
Acupuncture relieved cerebral ischemia–reperfusion (I/R) injury via suppressing endoplasmic reticulum (ER) stress. **a**, **b** The infarct volume of rats at 72 h after reperfusion in the four groups (I/R, I/R+ acupuncture, I/R+ acupuncture + 4-PBA, I/R+ acupuncture + tunicamycin (TM)) was detected by TTC staining. Brain infarct volume presented as a percentage of the infarct hemisphere. **c** The neurological deficits were determined by neurological score at 72 h after reperfusion. Neurological scores are presented as median (interquartile range). **d** Water content was determined. Results represent at least three independent experiments. **e** Blood brain barrier leakage was detected by Evans blue staining. Each experimental result was presented as mean ± standard deviation (n = 6). ^#^P < 0.05, ^###^P < 0.001 versus the indicated group

Next, to evaluate whether acupuncture protected against cerebral I/R injury via regulating ER stress, the rats were further administrated with ER stress inhibitor, 4-PBA, or ER stress activator, TM. As presented in Fig. 7a, b, acupuncture-induced decrease in infarct volume was further promoted by 4-PBA, but reversed by combination with TM. Additionally, as compared with I/R+ acupuncture group, we observed a downward trend in neurological score after administration with 4-PBA, while an upward trend in TM treatment group, although no statistical difference was obtained (Fig. 7c). As shown in Fig. 7d, the brain water content was detected. Acupuncture treatment markedly lowered the brain water content compared with I/R group. Likewise, combination with 4-PBA further lessened brain water content, while TM administration increased brain water content, as compared with acupuncture group. Consistent with water content in the brain, acupuncture-induced attenuation in BBB leakage assessed by EB staining was enhanced by 4-PBA, but counteracted by TM (Fig. 7e). Therefore, ER stress regulation participated in the protective mechanisms of acupuncture in cerebral I/R injury.

### Acupuncture inhibited autophagy via regulating ER stress in cerebral I/R injury

**Fig. 8:**
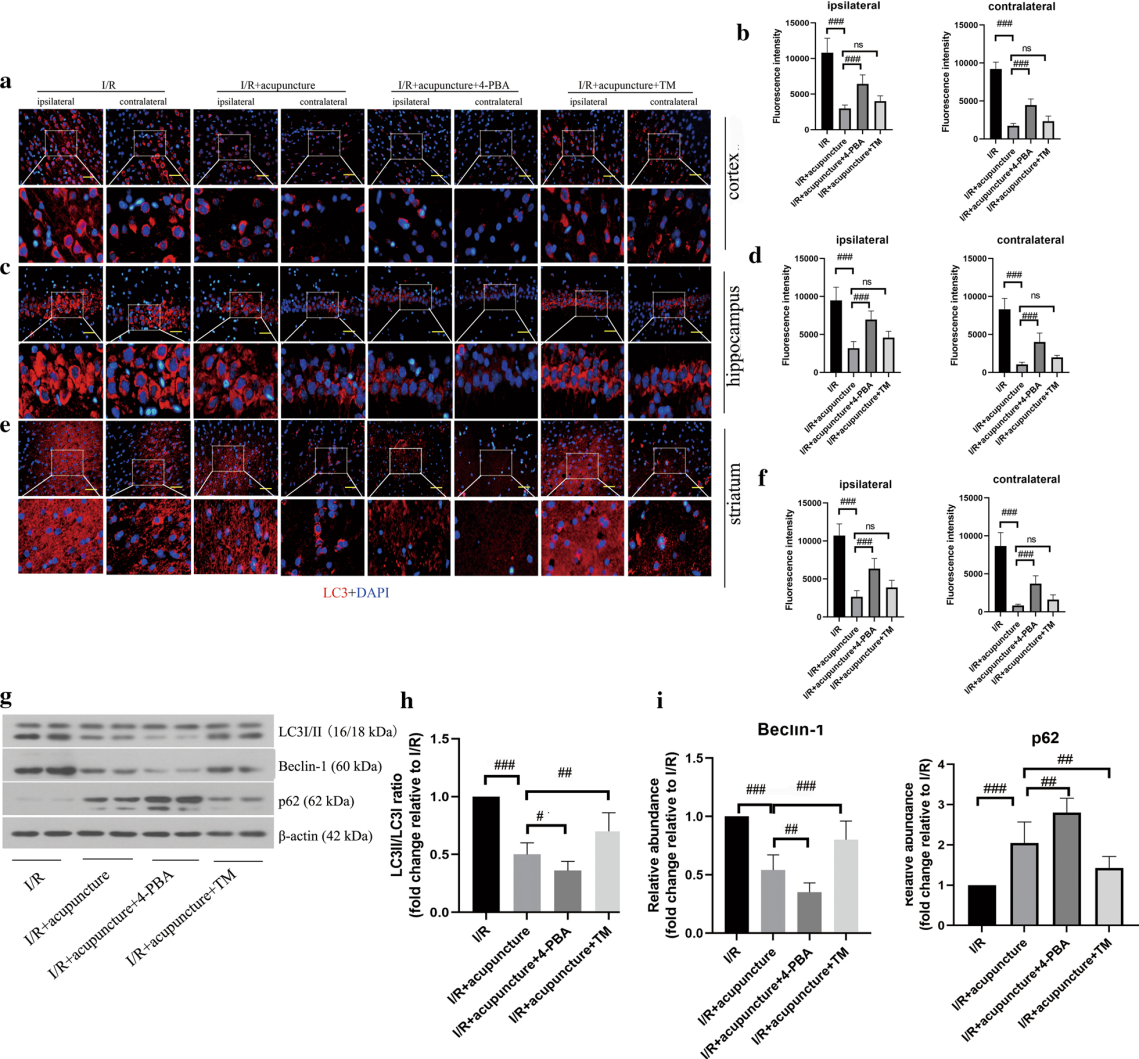
Acupuncture inhibited autophagy via regulating endoplasmic reticulum (ER) stress in cerebral ischemia–reperfusion (I/R) injury. The expression of LC3 (red) in contralateral and ipsilateral brain regions of cortex (**a**, **b**), hippocampus (**c**, **d**), and striatum (**e**, **f**) at 72 h after reperfusion was observed by immunofluorescence staining. The nuclei were counterstained with DAPI (blue). Scale bars, 50 μm. **g** The protein levels of LC3I/II, Beclin-1, and p62 in brain tissues were detected by Western blotting assay. β-actin was used as an internal control. **h**–**j** The relative abundance of the protein bands was calculated and shown. Results represent at least three independent experiments. Each experimental result was presented as mean ± standard deviation (n = 6). ^#^P < 0.05, ^##^P < 0.01, ^###^P < 0.001 versus the indicated group

Since ER stress is closely linked to autophagy and plays a vital role in the prognosis of cerebral I/R injury, we further explored whether acupuncture affected autophagy by regulating ER stress. As assessed by immunofluorescent staining and shown in Fig. 8a–f, LC3 expression in cortex, hippocampus and striatum of ipsilateral and contralateral cerebral hemisphere was decreased by acupuncture treatment compared with I/R injury group. Administration with 4-PBA further repressed LC3 expression in cortex, hippocampus and striatum as compared with I/R+ acupuncture group, whereas TM treatment presented the opposite tendency. Furthermore, the ratio of LC3II/I and Beclin-1 protein level were downregulated and p62 level was upregulated by acupuncture, which were strengthened by treatment with 4-PBA, but reversed by TM (Fig. 8g–j). Collectively, acupuncture intervention restrained autophagy through regulating ER stress in cerebral I/R injury.

### ER stress was involved in the neuroprotective effect of acupuncture against apoptosis in cerebral I/R injury

**Fig. 9:**
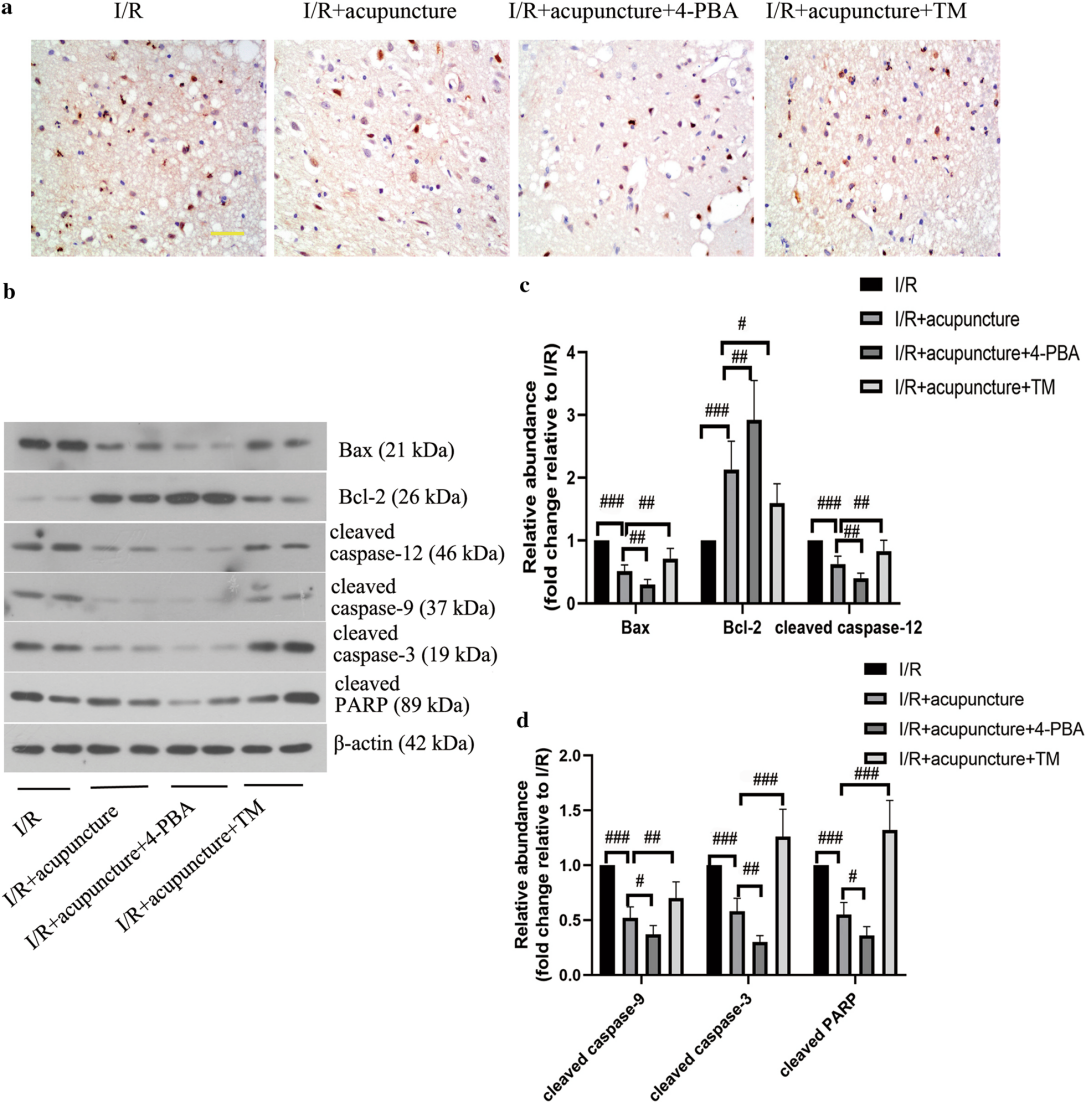
Endoplasmic reticulum (ER) stress was involved in the neuroprotective effect of acupuncture against apoptosis in cerebral ischemia–reperfusion (I/R) injury. Experiments were performed at 72 h after the reperfusion in the ipsilateral brain tissues. **a** The apoptosis in brain tissues was evaluated by TUNEL assay. Scale bars, 50 μm. **b** The protein levels of Bcl-2 Associated X Protein (Bax), B-cell lymphoma-2 (Bcl-2), cleaved caspase-12, cleaved caspase-9, cleaved caspase-3, and cleaved poly ADP-ribose polymerase (PARP) in brain tissues were detected by Western blotting assay. β-actin was used as an internal control. **c**, **d** The relative abundance of the protein bands was calculated and shown. Results represent at least three independent experiments. Each experimental result was presented as mean ± standard deviation (n = 6). ^#^P < 0.05, ^##^P < 0.01, ^###^P < 0.001 versus the indicated group

The apoptosis in brain tissues was assessed by TUNEL assay. As illustrated in Fig. 9a, positive apoptosis cells were observed in I/R injury group, whereas treatment with acupuncture restrained MCAO-induced apoptosis in brain tissues. Administration with 4-PBA enhanced the protective effect of acupuncture against apoptosis, however, TM treatment counteracted the anti-apoptosis effect of acupuncture. Moreover, acupuncture intervention downregulated the protein levels of Bax, cleaved caspase-12, cleaved caspase-9, cleaved caspase-3, and cleaved PARP, while upregulated Bcl-2 level compared with I/R group. Acupuncture-induced the above changes were further reinforced by combination with 4-PBA, but counteracted by TM administration (Fig. 9b–d). Therefore, acupuncture treatment suppressed apoptosis via inhibiting ER stress in cerebral I/R injury.

## Discussion

Ischemic stroke is a kind of cerebrovascular disease with limited therapy options, which severely harms human health. It has been reported that about 4.4 million patients died from ischemic stroke annually worldwide (Zheng et al. 2014) and majority of survivals lost labor ability, with high disability rate. At present, the annual healthcare costs for ischemic stroke are huge, which brings the serious burden on patients. Numerous studies revealed that acupuncture intervention could exert neuroprotective effects against cerebral I/R injury (Long et al. 2019; Zhang et al. 2018). However, the protective mechanisms of acupuncture on ischemic stroke have not be fully understood. In the present study, we demonstrated that acupuncture alleviated cerebral I/R injury via suppressing ER stress, autophagy and apoptosis. Acupuncture repressed MCAO-induced ER stress via PERK, IRE1, and ATF-6 pathways. In addition, inhibiting ER stress by 4-PBA could further repress autophagy and apoptosis, which strengthened the protective effects of acupuncture. While activating ER by TM enhanced autophagy, apoptosis and counteracted the beneficial effects of acupuncture. Our results shed light on the novel mechanisms of acupuncture in clinic treatment for ischemic stroke.

Scalp acupuncture therapy is an effective method to cure diseases via stimulating head acupoints. The effectiveness of scalp acupuncture in the treatment of stroke had been affirmed in the ancient China. In recent years, a lot of researches have confirmed that scalp acupuncture can improve motor dysfunction of stroke patients, and scalp acupuncture has been widely applied to clinical treatment of stroke (Wang et al. 2010, 2017, 2019). In this study, Baihui and Qubin acupoints were selected based on the experience from predecessors and lengthy clinical practices of our research group. Baihui acupoint belongs to Du Meridian, which can dispel wind, refresh mind, and clear heat for resuscitation. Qubin acupoint belongs to foot Shaoyang gallbladder meridian and is the junction of Foot-Taiyang and Foot-Shaoyang. According to our results, scalp electroacupuncture at Baihui and Qubin acupoints remarkably reduced infarct volume and alleviated neural functional injury of stroke rats, however sham acupuncture did not show any therapeutic benefit. Thus, acupuncture at Baihui and Qubin acupoints has a reliable curative effect on stroke. Next, we will focus on the potential molecular mechanisms of acupuncture in ischemic stroke.

ER stress is one of the pathogenetic mechanisms of cerebral I/R injury. For example, ER stress was verified to be involved in Reticulon Protein 1-C-mediated cerebral I/R injury in MCAO rats (Gong et al. 2017). Nan et al. demonstrated that inhibiting ER stress was the mechanism of cilostazol relieving cerebral I/R injury-induced endothelial cell damage (Nan et al. 2019). Therefore, ER stress inhibition can be an effective therapeutic strategy for cerebral I/R injury. ER stress is a process that wipes off the unfolded/misfolded proteins to keep cellular homeostasis, which is known as UPR. PERK, IRE1, and ATF6 are three UPR signaling sensors, the activation of which indicates UPR. GRP78 binds to these proteins to suppress their activation under normal conditions, whereas in response to ER stress, GRP78 converts to bind to unfolded/misfolded proteins, which result in the activation of PERK, IRE1, and ATF6 (Bertolotti et al. 2000; Lai et al. 2007). In this study, we found that MCAO led to obvious increase in GRP78, ATF-6, and phosphorylation of PERK and IRE1 in both ipsilateral and contralateral brain tissues, suggesting the activation of ER stress. However, acupuncture treatment significantly reversed the increased expressions of these proteins to restrain ER stress. Moreover, the activation of PERK can further promote the phosphorylation of eIF2α and enhance the expression of ATF-4, which finally results in the increased transcription of pro-apoptotic protein CHOP (Feng et al. 2017). According to our results, MCAO led to hyper-phosphorylation of eIF2α, and raised ATF-4 and CHOP levels, which was effectively suppressed by acupuncture intervention. Additionally, the neuroprotective effects of acupuncture were intensified by combination with ER stress inhibitor, 4-PBA, but counteracted by TM-mediated ER stress activation. Therefore, our findings proved that acupuncture treatment protected against cerebral I/R injury via suppressing ER stress.

Autophagy is a biological process controlled by autophagy-related genes, which degrades impaired organelles and macromolecular substances via lysosome. Currently, there are contradictory views on the effect of autophagy on ischemic stroke. Sheng et al. showed that ischemic preconditioning remarkably mitigated cerebral I/R injury through activating autophagy, while autophagy inhibitor 3-MA restrained the neuroprotective effect of ischemic preconditioning (Sheng et al. 2010). Carloni et al. also demonstrated that autophagy was activated in hypoxic-ischemic brain damage, and autophagy activator rapamycin treatment further inhibited apoptosis and attenuated brain injury (Carloni et al. 2008). While some research suggested that autophagy was harmful in cerebral I/R injury and acupuncture-mediated autophagy suppressing could reduce infarct volume and lessen neurological injury (Wu et al. 2015). Our results were consistent with the latter, acupuncture offered protection by suppressing autophagy in cerebral I/R injury as evidenced by decreased LC3II/I ratio and Beclin-1 level.

Increasing evidence has suggested that autophagy can be induced by ER stress (Song et al. 2018, 2017). It has been shown that autophagy was triggered by ER stress through upregulating Atg12 and the conversion of LC3I to LC3II in mouse embryonic fibroblasts (Kouroku et al. 2007). In mammalian cells PERK and IRE1 have been confirmed to mediate ER stress-induced autophagy (Kouroku et al. 2007; Ogata et al. 2006). Autophagosome formation was induced in SK-N-SH cells exposed to ER stress via IRE1-JNK pathway (Ogata et al. 2006). In addition, PERK/eIF2α pathway has been demonstrated to associate ER stress with autophagy in murine embryonic fibroblasts (Fujita et al. 2007). In the present study, acupuncture-induced autophagy inhibition in cerebral I/R injury was significantly reversed by ER stress activator TM, but enhanced by ER stress inhibitor 4-PBA. Therefore, acupuncture restrained cerebral I/R injury-induced autophagy via inhibiting ER stress.

Currently, much attention has been paid to ER stress-triggered apoptosis during cerebral I/R injury (Gong et al. 2017; Qiu et al. 2017). ER stress-related caspase 12 and CHOP proteins participate in apoptosis regulation (Kamarehei et al. 2019). It has been shown that the activation of PERK-eIF2α-ATF4 pathway results in increased CHOP expression that up-regulates Bax/Bcl-2 ratio and finally causes caspase-3 activation (Cai et al. 2015). Previous research indicated that the activation of ER-specific caspase-12 can trigger the following activation of caspase-9 and caspase-3, which leads to an apoptotic caspase cascade (Kim et al. 2019). Our findings indicated that acupuncture treatment alleviated cerebral I/R injury-induced apoptosis in brain tissues via down-regulating Bax/Bcl-2 ratio, and preventing caspase-12, caspase-9, and caspase-3 activation, which was enhanced by 4-PBA, but reversed by TM. Therefore, acupuncture relieved cerebral I/R-induced apoptosis via regulating ER stress.

## Conclusion

**Fig. 10:**
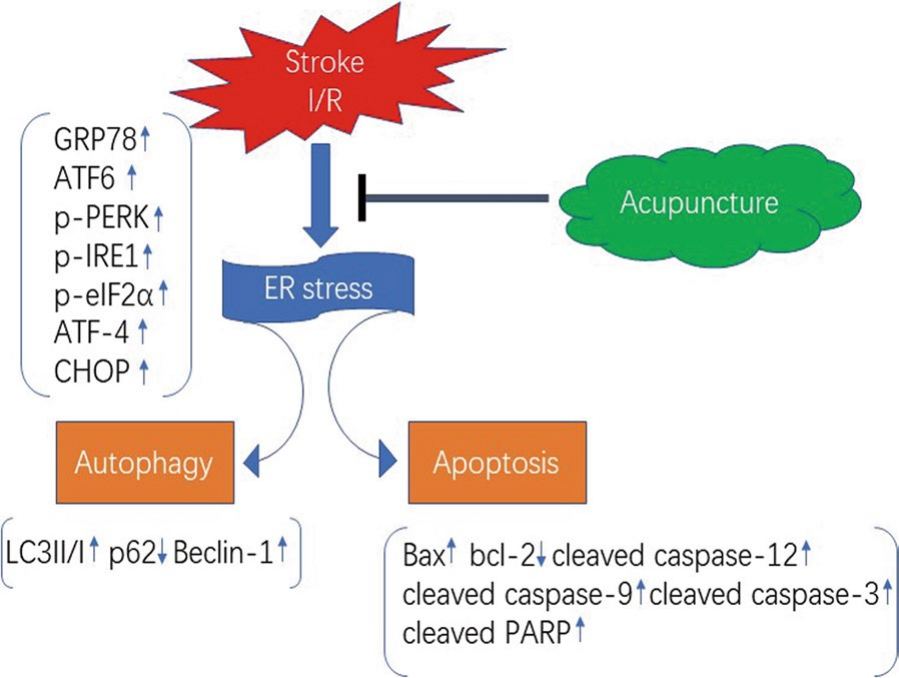
A simple diagrammatic schema. Acupuncture alleviates cerebral ischemia–reperfusion (I/R) injury-induced activation of endoplasmic reticulum (ER) stress as evidenced by inhibiting the expression of glucose-regulated protein 78 (GRP78), transcription factor 6 (ATF-6), transcription factor 4 (ATF-4), C/EBP-homologous protein (CHOP), and phosphorylation of PKR-like ER kinase (PERK), inositol-requiring protein 1 (IRE1), and α unit of eukaryotic initiation factor 2 (eIF2α), which subsequently restrained ER-stress dependent autophagy via down-regulating LC3II/I ratio and Beclin-1 levels and up-regulating p62 level. Acupuncture also suppressed ER-stress-mediated apoptosis via decreasing Bcl-2 Associated X Protein (Bax), cleaved caspase-12, cleaved caspase-9, cleaved caspase-3, cleaved poly ADP-ribose polymerase (PARP) expression, and increasing B-cell lymphoma-2 (Bcl-2) expression

Taken together, our findings suggest that acupuncture alleviates cerebral I/R injury by inhibiting ER stress via PERK, IRE1, and ATF6 signaling pathways. Moreover, acupuncture treatment suppressed ER-stress dependent autophagy and apoptosis during cerebral I/R injury. A simple diagrammatic schema is shown in Fig. 10. Therefore, our results shed new light on the protective mechanisms of acupuncture in the treatment of ischemic stroke.

## Supplementary information

**Additional file 1: Fig. S1.** Effect of acupuncture or sham acupuncture on ER stress triggered by cerebral I/R injury.

## Data Availability

The datasets used or analyzed during the current study are available from the corresponding author on reasonable request.

## Abbreviations

I/R: Ischemia–reperfusion
ER: Endoplasmic reticulum
MCAO: Middle cerebral artery occlusion
UPR: Unfolded protein response
PERK: PKR-like ER kinase
IRE1: Inositol-requiring protein 1
ATF-6: Activating transcription factor 6
GRP78: Glucose-regulated protein 78
CCA: Common carotid artery
ECA: External carotid artery
ICA: Internal carotid artery
TM: Tunicamycin
TUNEL: Terminal deoxynucleotidyl transferase-mediated nick end labeling
