# Supplementary figures and images for: Acupuncture protects against cerebral ischemia–reperfusion injury via suppressing endoplasmic reticulum stress-mediated autophagy and apoptosis
Source: Mol Med. 2020 Nov 10;26:105. doi: 10.1186/s10020-020-00236-5 (PMC7653860; doi:10.1186/s10020-020-00236-5)

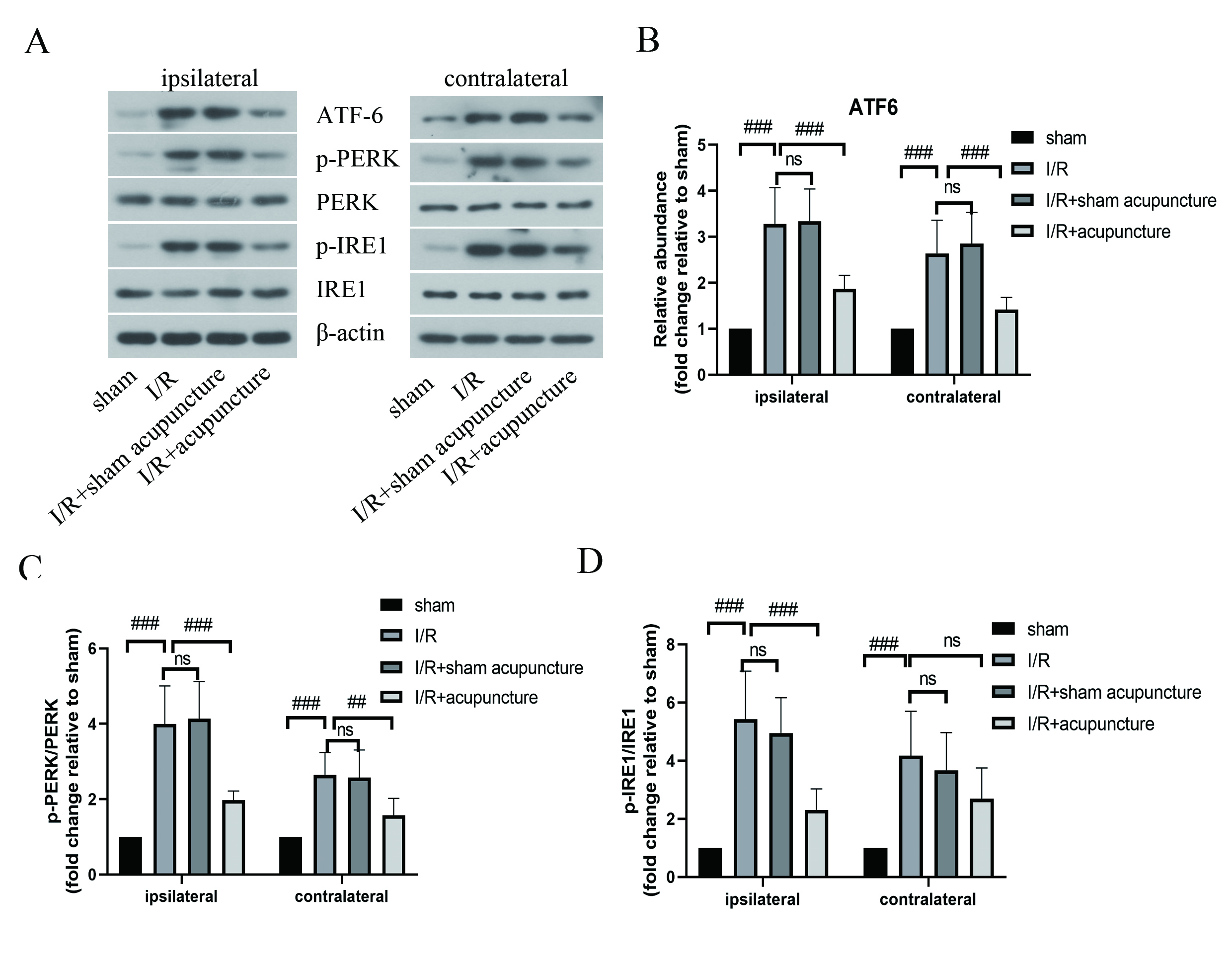

Supplement: Supplementary file 1 — Additional file 1: Fig. S1. Effect of acupuncture or sham acupuncture on ER stress triggered by cerebral I/R injury. [file 10020_2020_236_MOESM1_ESM.jpg]
